# Every prescription counts: rethinking outpatient antibiotic stewardship in Abu Dhabi, United Arab Emirates

**DOI:** 10.3389/fpubh.2026.1761960

**Published:** 2026-02-02

**Authors:** Kanika Vats, Samar Alnasir, Rahaf Ajaj

**Affiliations:** 1 Directorate of Research and Innovation, TASNEEF (Emirates Classification Society), Abu Dhabi, United Arab Emirates; 2 Department of Public Health, Abu Dhabi University College of Health Sciences, Abu Dhabi, United Arab Emirates; 3 Department of Pharmacy, Medeor 24x7 Hospital, Abu Dhabi, United Arab Emirates; 4 Department of Environmental Health and Safety, Abu Dhabi University College of Health Sciences, Abu Dhabi, United Arab Emirates

**Keywords:** antibiotic, antibiotic stewardship program (ASP), antimicrobial resistance (AMR), clinical outcomes, Eastern Mediterranean region (EMR), outpatient settings, surveillance, United Arab Emirates (UAE)

## Abstract

Antimicrobial resistance (AMR) is a persistent global health challenge that is associated with morbidity, mortality, and healthcare costs. In most outpatient (OP) settings, such as emergency rooms, primary and specialty care clinics, and dental clinics, antibiotics are often inaccurately prescribed due to patient expectations, empirical decision-making, and limited access to diagnostic data. Despite existing national frameworks, surveillance systems, and stewardship initiatives, resistance among key pathogens in the United Arab Emirates (UAE), a part of the World Health Organization’s (WHO’s) Eastern Mediterranean Region (EMR), is increasing. Many OP settings are underdeveloped, as most efforts focus on hospital inpatients, leading to inappropriate antibiotic use, the promotion of resistant pathogens, and an increased risk of community-acquired infections. Intensifying antimicrobial stewardship (AMS) practices in OP settings brings an opportunity to enhance patient care, limit unnecessary prescriptions, prevent adverse reactions, and reduce healthcare costs. This perspective provides insights into regional AMR trends in the EMR, reviews existing governance, regulatory frameworks, and surveillance systems of the UAE, and identifies gaps with OP stewardship practices in the Emirate of Abu Dhabi. It recommends a multi-level framework to support optimisation of OP antibiotic use, guided by regional trends, current practice, and lessons learnt from the COVID-19 pandemic. By adopting this framework, current stewardship efforts can be strengthened, responsible antibiotic use promoted, and surveillance improved, progressing towards a coordinated, multi-sector strategy to preserve antimicrobial efficacy and sustain long-term progress against resistance.

## 1 Introduction

“Every antibiotic prescription matters, and each compromised selection worsens an ongoing crisis we cannot afford to ignore.”

Antimicrobial resistance (AMR) is the most persistent threat to global health in the 21st century, leading to a substantial burden on healthcare systems (1). In 2019, due to overuse and misuse of antibiotics, bacterial AMR directly caused an estimated 1.27 million deaths and contributed to nearly 5 million more, contributing to the emergence of drug-resistant pathogens (2). By 20,250, AMR may result in annual global gross domestic product (GDP) loss of US$1–34 trillion and healthcare costs of US$1 trillion (3).

Global surveillance indicates widespread antibiotic resistance, particularly among Gram-negative pathogens, with regional variations. Low and middle-income nations and regions with weaker health systems are disproportionately affected, and countries with limited surveillance frequently report higher levels of resistance (4).

Most of the antibiotic use occurs in outpatient (OP) settings, such as emergency departments (EDs), urgent care centres, dental clinics, primary care, speciality and subspeciality clinics, and community pharmacies. In the US, OP care accounts for about 60% of all antibiotic spending (5), while in other developed nations, 80–90% of antibiotic use occurs outside hospitals (6). OP prescribing is particularly susceptible to misuse due to patient expectations, “just in case” empirical prescriptions, and limited access to microbiological data across decentralised systems.

Existing outpatient antimicrobial stewardship programs (OP-ASP) encourage the careful use of antibiotics, thereby enhancing patient outcomes and combating antibiotic resistance (7). However, tracking prescription trends and utilising antibiotic usage and resistance data usually guide stewardship initiatives and policy development (8).

The COVID-19 pandemic has led to inappropriate antibiotic use both in hospitals and OP settings (4, 9–12), highlighting healthcare system vulnerabilities during the public health crises.

The United Arab Emirates (UAE) is not immune to these evolving resistance trends, with available evidence indicating widespread and increasing AMR in clinical settings. In 2023, the prevalence of multidrug-resistant, extensive drug-resistant, and pan-drug resistant for AMR priority pathogens was reported at 37.7, 3.9, and 0.4%, respectively (11).

Given rising regional resistance and still-maturing OP-ASP practices in the Emirate of Abu Dhabi, this perspective article, guided by existing governance and surveillance frameworks, current stewardship practices, and lessons learned from the COVID-19 pandemic, proposes a multisectoral framework to strengthen ongoing efforts. The current question is not whether the change is required, but how quickly it can be implemented to accelerate progress.

## 2 Antimicrobial resistance in the eastern mediterranean region

The United Arab Emirates (UAE), part of the World Health Organization’s (WHO) Eastern Mediterranean Region (EMR) (13), provides an important context for thoughtful consideration of the rise of AMR. Regional trends help frame national data, highlight shared challenges, and offer lessons the UAE can apply to strengthen its own strategies.

In 2023, 16 of 21 EMR countries reported data to the global AMR surveillance system. Around 30% of infections were resistant to commonly used antibiotics; nearly one in three infections had high levels of resistance; and methicillin-resistant *Staphylococcus aureus* (MRSA) accounted for half of bloodstream infections caused by *Staphylococcus aureus*, nearly twice the global average. Fluoroquinolone resistance in *Neisseria gonorrhoeae* was widespread [75%], and emerging ceftriaxone resistance threatens one of the last reliable treatment options (4).

Surveillance capacity remains inconsistent; only about half of EMR countries have fully implemented core surveillance components, which is below the global average. Uneven surveillance and underreporting may lead to misinterpretation of AMR burden, which can affect benchmarking, regional comparisons, planning, and prioritization of OP-ASP initiatives. Despite this, the EMR provided the highest-quality, most complete data across all regions (4).

Key pathogens such as *E. coli* and *K. pneumoniae* exhibit high levels of multidrug resistance. While third-generation cephalosporins and carbapenems remain effective against *Salmonella*, fluoroquinolone resistance is high. *Shigella* shows significant ceftriaxone resistance [~57%] but very low resistance to azithromycin [1%] (4).

Overall, resistance in the EMR is concerning, i.e., *E. coli* resistance to third-generation cephalosporins is 65%, MRSA is 50.3%, and carbapenem-resistant *Acinetobacter* is 66.5%, all above global averages (4). These findings highlight both advancement and current gaps. Strengthened surveillance, standardized policies, and coordinated action are critical to prevent AMR from escalating further.

## 3 Governance, regulations, and surveillance in the UAE

### 3.1 National oversight and framework

To address the growing threat of AMR, the UAE developed a National Strategy and Action Plan (14, 15). It established a nationwide surveillance system covering inpatient and outpatient settings across public and private healthcare facilities. However, the country still lacks a national AMR reference laboratory (11), and surveillance does not yet cover the entire health sector.

In 2015, a Higher Committee for AMR with several technical subcommittees was established (11, 16). Since 2018, the nation has been contributing data to the Global AMR Surveillance System (GLASS) (11), with each surveillance site designating a focal point to collect and report data in accordance with GLASS guidelines (17).

Most microbiology laboratories use automated systems to support consistent phenotypic AMR reporting (16). While international antimicrobial susceptibility testing (AST) guidelines provide the universal reference, UAE laboratories may also follow national guidance to align local reporting practices with stewardship efforts. Currently, there are no comprehensive national AST guidelines, and laboratories rely on internationally accepted standards and, when needed, alternative global breakpoints (11).

Additionally, the UAE continues to invest in professional collaboration and training. For instance, the Ministry of Health’s 8th UAE International Conference on AMR, which convened over 600 healthcare professionals (18).

The UAE has introduced national empirical treatment guidelines for selected infections, such as pediatric UTIs and skin and soft tissue infections, helping standardize evidence-based prescribing and strengthen AMS efforts (19, 20).

Overall, while significant progress towards building a sustainable AMR framework is evident, further expansion of surveillance systems and standardized testing practices is desired to support reliable data, enhance stewardship, and advance a unified national AMR response.

### 3.2 Distribution of surveillance sites across the UAE

The UAE first established its AMR surveillance system at the subnational level in 2010, starting in the Emirate of Abu Dhabi with 22 sites. In 2015, the system was expanded nationwide through collaboration between the Ministry of Health and Prevention, the Ministry of Presidential Affairs, the Dubai Health Authority, the Department of Health – Abu Dhabi, and the Abu Dhabi Public Health Center (11).

As of December 2023, the UAE National AMR Surveillance System operates as a laboratory-based network of 44 clinical microbiology laboratories across seven Emirates, supporting 318 surveillance sites, including 87 hospitals and 231 clinics/centers (11). However, the system remains skewed toward inpatient settings: 87 of 151 hospitals [57.6%] participate, while OP clinics/centers are underrepresented, with only 231 of approximately 2,730 sites included [8.5%] (11).

Surveillance coverage is uneven across the Emirates. Abu Dhabi, with 36 hospitals and 104 clinics/centers supported by 17 laboratories, leads participation. Dubai has fewer hospitals [28] and clinics [64], but the highest number of laboratories [19], highlighting potentially enhanced laboratory-based surveillance capacity. Smaller Emirates like Sharjah and Ras Al Khaimah have reasonable coverage [7 hospitals and 21 clinics each], but very few laboratories [2–3]. In contrast, Fujairah, Ajman, and Umm Al Quwain have nominal participation, with only 1 laboratory in each Emirate (11).

Such uneven distribution is susceptible to introducing blind spots and restricting inclusive epidemiological analyses. Moreover, reported resistance data are mainly from inpatient isolates, which may not fully reflect OP practice patterns. Therefore, strategic expansion of surveillance sites in underserved areas is recommended to ensure comprehensive, actionable data collection and strengthen national AMR policy development.

### 3.3 National AMR status

As of 2023, published reports have shown an increasing resistance across several priority pathogens (11, 21), aligning with the resistance patterns reported for the broader EMR (4). Resistance in *E. coli* ranged from 1.0% for carbapenems (meropenem) to 63% for aminopenicillins (ampicillin); *K. pneumoniae* showed resistance from 4.4% for carbapenems (meropenem) to 30% for cefuroxime; *Salmonella* spp. demonstrated 54% resistance to ciprofloxacin, and MRSA accounted for 38% for all isolates (11).

Data from Abu Dhabi and other Emirates highlight emerging resistance patterns, while also reflecting gaps in current surveillance coverage (11, 21). Although the existing national surveillance system provides robust AMR data, national-level antimicrobial consumption data remain limited for all clinical settings, despite the well-established association between antimicrobial use and AMR rates.

Overall, the UAE has made significant progress in governance, regulatory frameworks, and surveillance; however, current mechanisms do not consistently influence OP-ASP, uneven coverage, and limited standardization may reduce data comparability and stewardship effectiveness.

## 4 Outpatient antibiotic stewardship (asp) in Abu Dhabi

While UAE-wide AMS initiatives provide a national landscape, existing OP stewardship initiatives in the Emirate of Abu Dhabi serve as a focused example of early-stage efforts to improve antibiotic use and strengthen local surveillance, thereby contributing to broader national efforts to address AMR.

### 4.1 Implementation and monitoring

In the Emirate, the Antimicrobial Stewardship Program (ASP) is mandatory across hospitals and one-day surgery centers providing inpatient care, outpatient, emergency, palliative, and rehabilitation services, and is supported by annual reporting of ASP interventions, quantitative and qualitative indicators related to antimicrobial prescribing practices, resistance trends, and quality of care. The mandate encourages responsible prescribing, consistent monitoring, and stronger accountability (22–24) to facilitate ongoing efforts to combat AMR.

While hospital-based ASPs are well developed and collaboratively monitored by the Pharmacy and Therapeutics Committee and ASP teams, their focus is predominantly inpatient. As such, many OP settings (25), including dental clinics/centers, primary care and specialty clinics/centers, telemedicine providers, mobile health units, and other health service providers, have yet to be fully integrated into the ASP framework.

Similarly, OP pharmacies, including community, retail, hospital-based OP, and ambulatory pharmacies, are partially integrated into ASP. Some categories (e.g., hospital, one-day surgery, ambulatory, 24-h, remote-area pharmacies) are allowed to dispense broad-spectrum antibiotics to ensure accessibility (26), while others are restricted to regulate regional AMR efforts (27–29). In addition, the extent to which telemedicine providers, online pharmacies, or home-delivery platforms are regulated for prescribing antibiotics and tracking their use is unclear, which may increase the risk of contributing to the rise of AMR.

While Abu Dhabi has made considerable progress in strengthening ASP efforts, there are persisting gaps across the broader OP settings. Expanding ASP use across diverse OP settings, including pharmacies, without compromising accessibility or stewardship, is crucial. Doing so will support a system-wide AMS approach and help maintain long-term momentum against AMR.

### 4.2 Managing antibiotic use in outpatient care

Antibiotics are classified as “Prescription Only Medicines” in the Emirate, requiring pharmacists to follow defined dispensing guidelines, counsel patients on appropriate use, and discourage self-medication (30).

While audits monitor compliance, vigilance is needed, as patient antibiotic access varies despite these controls. Dispensing of broad-spectrum antibiotics is linked to health insurance approvals, limiting their dispensing by authorized pharmacies (28, 29). Yet challenges remain for patients paying out of pocket, who may obtain prescribed antibiotics from pharmacies outside these controls or receive them directly from pharmacy stock, creating regulatory gaps. These issues highlight that effective AMS must address both prescribing practices and dispensing restrictions.

**Figure 1:**
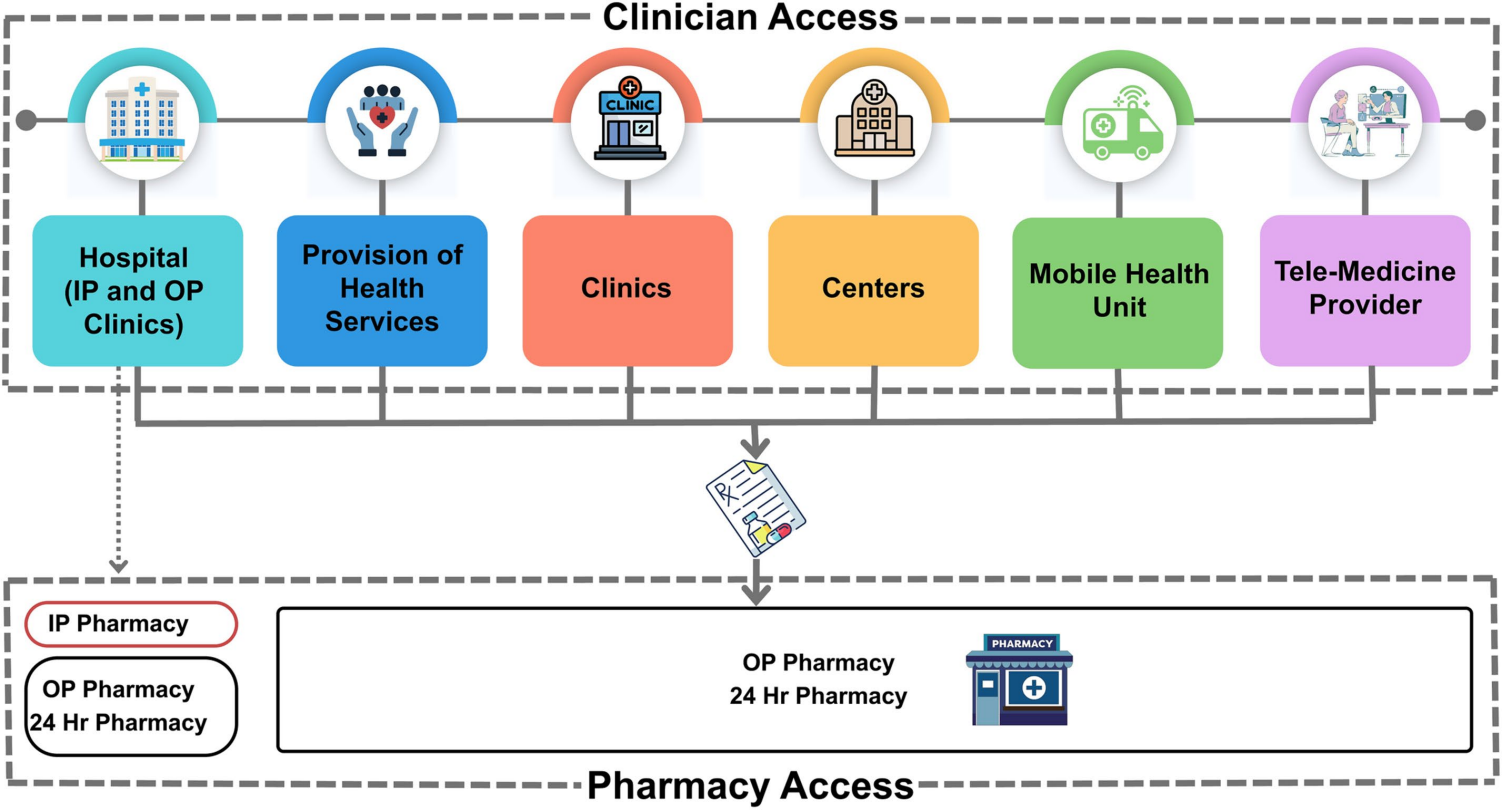
Patient pathways to antibiotic access in outpatient settings. For illustration purposes, the categorization of outpatient settings is based on the DoH facility licensing system, grouped by classification types (25) with clinical practice privileges defined according to DoH-authorized authorizations: Hospital: Includes specialized hospital, general hospital, rehabilitation hospital, and nursing home. OP are specialized clinics within hospitals. Provisions of Health Services: Encompasses home care services, patient escort, healthcare management, medical transport (ambulance, air ambulance, MTU), and medical staffing (employment and transfer). Clinics: Includes school clinics, first aid posts, general medical clinics, specialized medical clinics, general dental clinics, and specialized dental clinics. Centers: Includes dental centers, medical centers, primary healthcare centers, and one-day surgery centers. (Additional facilities such as fertilization centers, rehabilitation centers, and dialysis centers are grouped under “Centers” for figure clarity, though they exist as independent facility types in the DoH Shafafiya listing). Mobile Health Unit: Includes medical laboratories, dental services, first aid posts, general/specialized clinics, and rehabilitation services. DoH, department of health; Hr, hour, IP, inpatient; MTU, medication transportation unit; OP, outpatient.

Figure 1 shows how prescriptions generated across various OP settings flow into pharmacies. Only hospital inpatients are required to collect medications from the inpatient pharmacy at discharge; all other patients may use any OP pharmacy of their choice. This highlights that the most critical intervention point for AMS is when a prescription is issued. Once an antibiotic is prescribed, inappropriate use becomes far more difficult to prevent, highlighting the importance of early action at the prescribing stage.

Additionally, most OP antibiotics are dispensed in their original manufacturer packaging through the health insurance system, rather than in quantities tailored to the prescribed duration. Unit-of-use dispensing is limited to narcotic and psychotropic medications. This practice can lead to leftover antibiotics, which patients may use later or share with others (31) or dispose of improperly, behaviors that contribute to AMR (32, 33).

These challenges are made worse when clinicians feel pressured by patients or worried parents who want quick relief, and, to maintain patient satisfaction, prescribe antibiotics unnecessarily, even for viral infections (34). Therefore, AMS efforts are required beyond pharmacy-level controls and adopting a system-wide approach. Appropriate antibiotic use must be ensured through active monitoring of prescribing practices, understanding patient pathways, and addressing prescribing and dispensing gaps.

### 4.3 Access to centralized medical records to support AMS in outpatient care

To standardize patient demographics and clinical data, Emirate has a centralized health information exchange (Malaffi) across EDs, OP clinics, and pharmacies, and as per regulatory mandate, facilities are required to maintain appropriate records for care delivery and medication dispensing (35–39). However, the system is yet to reach maturity, particularly in terms of full accessibility and real-time data availability across all healthcare providers.

For effective AMS interventions, reliable access to both lab results, especially microbiology reports, and patients’ prescription and allergy histories is essential for initiating evidence-based treatment and ensuring continuity of care. Since microbiology reports indicate infection and sensitivity profiles, and prescription records show prior antibiotic use, clinicians are better equipped to avoid duplicate therapy, inappropriate therapy, or ineffective treatments. Therefore, having access to centralized medical records with this information at the time of the patient encounter can improve prescribing accuracy, support care planning, and reinforce overall OP-ASP efforts.

### 4.4 Optimizing antimicrobial use in OP settings: a multi-level approach

With an understanding of current AMS initiatives, rising resistance trends, existing clinical practices, and challenges related to the maturity of AMS systems, a multi-level approach involving multiple sectors and stakeholders is recommended, as outlined below.

#### 4.4.1 System-level integration

A system-level reform that embeds AMS into the health infrastructure will enhance policy alignment, strengthen regulatory oversight, and promote system-wide accountability. To support this reform, key initiatives may include expanding surveillance sites to track antibiotic use and resistance trends, integrating AMS-related quality indicators into facility licensing to impose compliance, incorporating AMS strategies into emergency response plans for infectious disease outbreaks (4, 11), and linking approvals for evidence-based broad-spectrum antibiotics with the payer system. As part of this linking process, approvals may be restricted to the required unit-of-use, and duplicate anaerobic therapy authorizations may be prevented, helping ensure clinical appropriateness, reducing misuse, strengthening stewardship efforts, and improving cost efficiency.

#### 4.4.2 Clinical optimization

Developing prescribing guidelines for specific clinical syndromes, in alignment with local disease patterns and patient risk factors, will support the selection of effective empiric therapy and reduce unnecessary broad-spectrum use. Targeted improvement can be achieved by identifying high-priority conditions in which clinicians often deviate from best practices (7). Additional strategies, such as prospective audits, evidence-based delayed prescribing, and patient follow-up, can further support rational antibiotic use, helping to reduce overprescribing, antibiotic resistance, adverse reactions, and costs. Engaging patients in care plans and treatment reviews further reinforces AMS practices, helping clinicians adhere to best practices and guide treatment therapy tailored to patient needs.

#### 4.4.3 Technology and analytics

Emerging technologies can help accelerate the maturity levels of the AMS program within the Emirate. Initiatives may include integrating key indicator alerts into e-prescribing systems, allowing centralized antibiotic tracking through Malaffi, implementing electronic approval workflows for restricted antimicrobials within health provider systems, and using AI-driven diagnostics, surveillance, and resistance forecasting to improve accuracy and support early intervention.

#### 4.4.4 Community and patient engagement

Since the community and patients are on the receiving end of the treatment cycle, actively involving patients and promoting public education are equally important. Campaigns to raise awareness of antibiotic resistance, pharmacist-led advice on appropriate use (7), gathering public input to guide policy, and patient monitoring to direct therapy modifications are a few examples of initiatives. Once the source of the infection has been identified, the seven-day follow-up window for insured outpatients provides an opportunity to review empirical therapy and switch to targeted treatment.

#### 4.4.5 Workforce development

Another key element of sustainable AMS is building a competent workforce. All newly recruited healthcare professionals should complete and document ASP orientation training within the first 3 months of employment (22). Creating accredited courses grounded in One Health principles (40), integrating AMS into academic curricula, hosting interdisciplinary workshops, and requiring AMS-focused CME for health professional licensing are among possible strategies.

#### 4.4.6 Research and compliance

Periodic regulatory compliance audits, pilot testing of low-cost AMS interventions, and financing clinical studies, such as those on treatments, diagnostics, or retrospective reviews, that produce evidence on what is safe, effective, and feasible in real-world settings to inform best practices, are some ways to improve evidence generation and accountability. To further encourage high-quality care, a Centre of Excellence program can be established to honour exceptional providers. These initiatives shall support evidence-based policymaking and promote continuous improvement.

**Figure 2:**
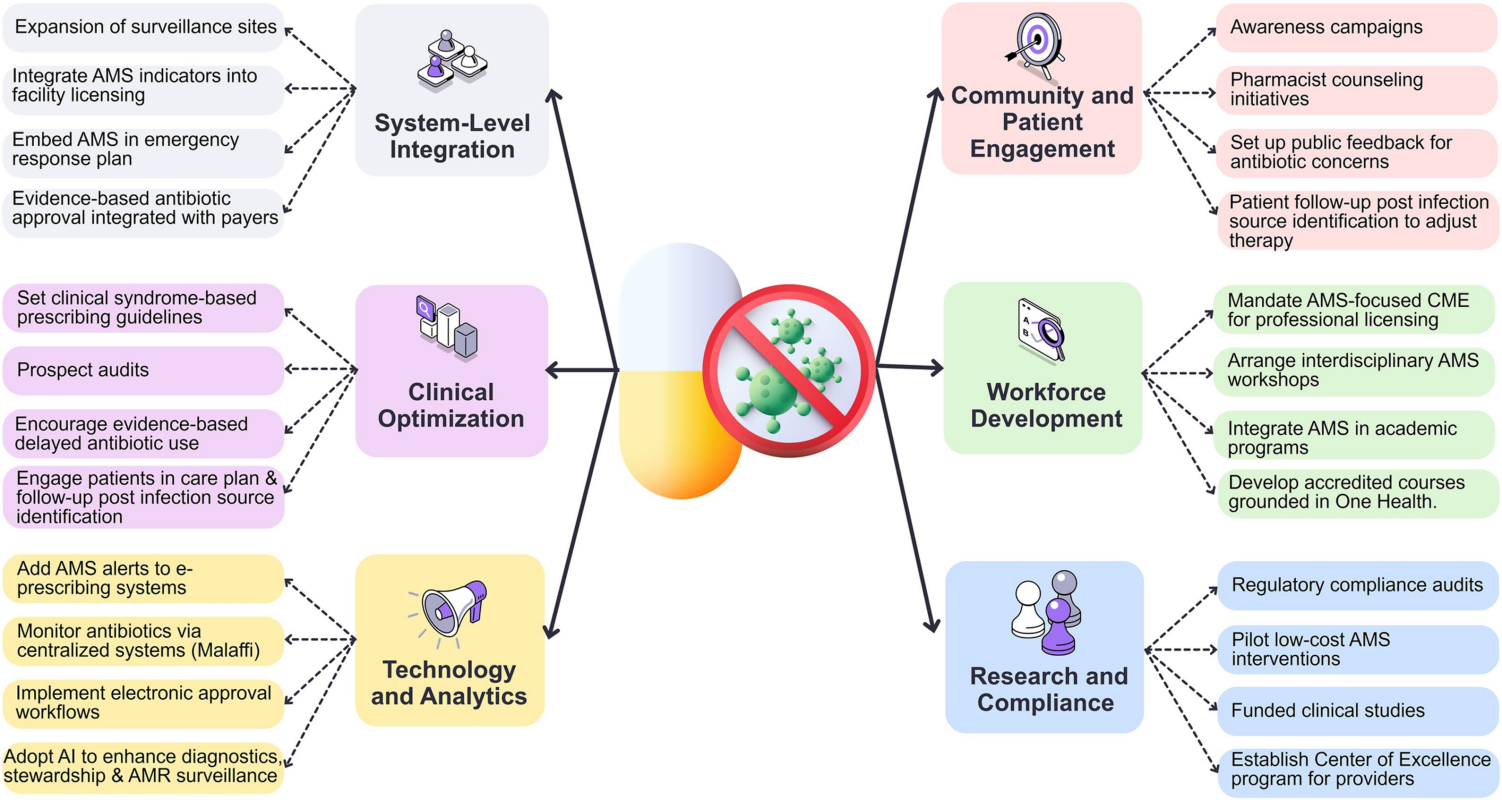
Multi-level framework for optimized antimicrobial stewardship in outpatient settings. “Payers” are health insurance coverage providers, and “Providers” are healthcare settings that deliver clinical care. AMS, antimicrobial stewardship; AMR, antimicrobial resistance; AI, artificial intelligence; CME, continuous medical education.

Figure 2 summarises this multi-level AMS strategy, organized around the six interconnected pillars described above, to optimize antimicrobial use and combat resistance. Applying this framework shall strengthen existing ASPs and provide a sustainable, scalable approach.

## 5 Discussion

Across the Gulf region, antibiotic prescribing in OP, including ED, is suboptimal (41). In the UAE, antimicrobial use and hospital-acquired infection rates align with regional and global trends, highlighting the need for stricter local guidelines to optimize use of Watch and Reserve category antibiotics, reducing prolonged post-surgical therapy (42) and limiting unnecessary prescriptions.

In EMR, *E. coli* is the most reported pathogen, followed by *K. pneumoniae*, and MRSA accounts for over half of bloodstream infections, nearly double the global average (4). In alignment, the UAE has designated *E. coli*, *K. pneumoniae*, and *Staphylococcus aureus*, among others, as AMR priority pathogens, stressing the need for targeted surveillance and stewardship practices (11).

In Abu Dhabi, stewardship efforts are more focused on hospitals, leaving OP settings less developed (43). Often, community *Clostridium difficile* infections have been linked to inappropriate OP antibiotic use (44), and, on the other hand, targeted stewardship interventions in primary care facilities have proven successful in reducing prescription rates and thereby associated costs (45). Therefore, expanding ASP to OP settings can improve patient care, limit community-level resistance, prevent adverse events (46, 47) and reduce healthcare expenditures.

In the current era of digitalization, digital tools can further support stewardship efforts, for example, a smartphone application equipped with local guidelines has shown improved OP prescribing (48). Despite existing regulations, in one survey, nearly one-third of UAE participants reported obtaining antibiotics without a prescription; therefore, raising public awareness about self-medication (34) and monitoring dispensing practices among pharmacies is also a need of the hour. Expanding surveillance sites in the Emirate and establishing a National Reference Laboratory would further enhance data quality and improve the detection of emerging strains (11).

Adopting a restricted authorization approach can help reduce leftovers, inappropriate use, and improper disposal among patients and communities, supporting environmental protection in line with the One Health approach. Pharmacists are key to OP-ASP, supporting improved antibiotic usage (49) and their impact can be enhanced through standardized training, updated curricula, and supportive regulations (50–53). However, despite 77% working in community settings, their contributions are often limited to dispensing due to outdated legislation and low remuneration (54).

The COVID-19 pandemic highlighted vulnerabilities in stewardship practices, contributing to rising levels of resistance (9, 21). Antibiotics in stockpiles are prone to misuse during crises and supply disruptions (55, 56). Therefore, integrating stewardship strategies into emergency response plans is crucial to safeguard rational antibiotic use.

This Perspective relies primarily on secondary data and existing surveillance systems that are predominantly inpatient-based, which may limit the direct applicability of some resistance trends to OP practice and highlight the need for expanded OP-specific surveillance.

In conclusion, OP-ASP provides a critical opportunity to optimize antibiotic use beyond hospitals. Implementing the proposed coordinated and multi-level approach will support sustainable stewardship efforts, promote responsible antibiotic use, and strengthen a resilient healthcare system. While focused on OP settings, this framework can also be adapted to enhance inpatient stewardship where needed.

## Data Availability

The original contributions presented in the study are included in the article/supplementary material, further inquiries can be directed to the corresponding author.
